# *N*-(4-Hy­droxy­phen­yl)-3,4,5-trimeth­oxy­benzamide

**DOI:** 10.1107/S1600536811040554

**Published:** 2011-10-08

**Authors:** Hyeong Choi, Yong Suk Shim, Byung Hee Han, Sung Kwon Kang, Chang Keun Sung

**Affiliations:** a Department of Chemistry, Chungnam National University, Daejeon 305-764, Republic of Korea; b Department of Food Science and Technology, Chungnam National University, Daejeon 305-764, Republic of Korea

## Abstract

In the title amide compound, C_16_H_17_NO_5_, the dihedral angle between the benzene rings is 71.59 (4)°. In the crystal, inter­molecular N—H⋯O and O—H⋯O hydrogen bonds link the mol­ecules into a two-dimensional array parallel to the *ab* plane.

## Related literature

For general background to tyrosinase and melanin, see: Kubo *et al.* (2000 ▶); Nerya *et al.* (2004 ▶). For the development of potent inhibitory agents of tyrosinase, see: Cabanes *et al.* (1994 ▶); Casanola-Martin *et al.* (2006 ▶); Thanigaimalai *et al.* (2010 ▶).
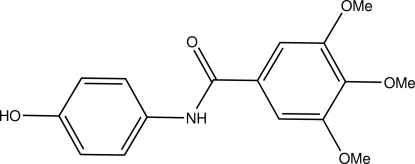


## Experimental

### 

#### Crystal data

C_16_H_17_NO_5_
*M* *_r_* = 303.31
Orthorhombic, 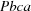

*a* = 10.4280 (5) Å
*b* = 13.4075 (6) Å
*c* = 21.5565 (8) Å
*V* = 3013.9 (2) Å^3^
*Z* = 8
Mo *K*α radiation
μ = 0.1 mm^−1^
*T* = 296 K
0.23 × 0.16 × 0.08 mm

#### Data collection

Bruker SMART CCD area-detector diffractometer
14730 measured reflections
3459 independent reflections
2086 reflections with *I* > 2σ(*I*)
*R*_int_ = 0.130

#### Refinement

*R*[*F* ^2^ > 2σ(*F* ^2^)] = 0.054
*wR*(*F* ^2^) = 0.134
*S* = 0.97
3459 reflections
207 parameters
H atoms treated by a mixture of independent and constrained refinement
Δρ_max_ = 0.23 e Å^−3^
Δρ_min_ = −0.29 e Å^−3^

### 

Data collection: *SMART* (Bruker, 2002 ▶); cell refinement: *SAINT* (Bruker, 2002 ▶); data reduction: *SAINT*; program(s) used to solve structure: *SHELXS97* (Sheldrick, 2008 ▶); program(s) used to refine structure: *SHELXL97* (Sheldrick, 2008 ▶); molecular graphics: *ORTEP-3* (Farrugia, 1997 ▶); software used to prepare material for publication: *WinGX* (Farrugia, 1999 ▶).

## Supplementary Material

Crystal structure: contains datablock(s) global, I. DOI: 10.1107/S1600536811040554/is2785sup1.cif

Structure factors: contains datablock(s) I. DOI: 10.1107/S1600536811040554/is2785Isup2.hkl

Supplementary material file. DOI: 10.1107/S1600536811040554/is2785Isup3.cml

Additional supplementary materials:  crystallographic information; 3D view; checkCIF report

## Figures and Tables

**Table 1:** Hydrogen-bond geometry (Å, °)

*D*—H⋯*A*	*D*—H	H⋯*A*	*D*⋯*A*	*D*—H⋯*A*
N9—H9⋯O17^i^	0.87 (2)	2.18 (2)	3.029 (2)	165.8 (17)
O16—H16⋯O8^ii^	0.88 (4)	1.84 (4)	2.710 (2)	172 (3)

Symmetry codes: (i) 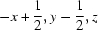; (ii) 
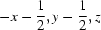
.

**Fig. 1.**
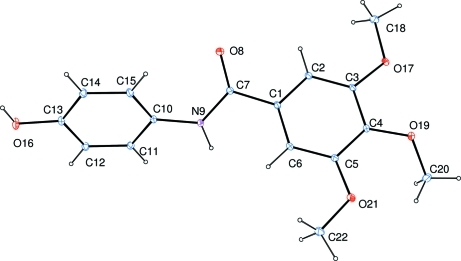
A molecular view of the title compound, showing the atom-numbering scheme and 30% probability ellipsoids.

**Fig. 2.**
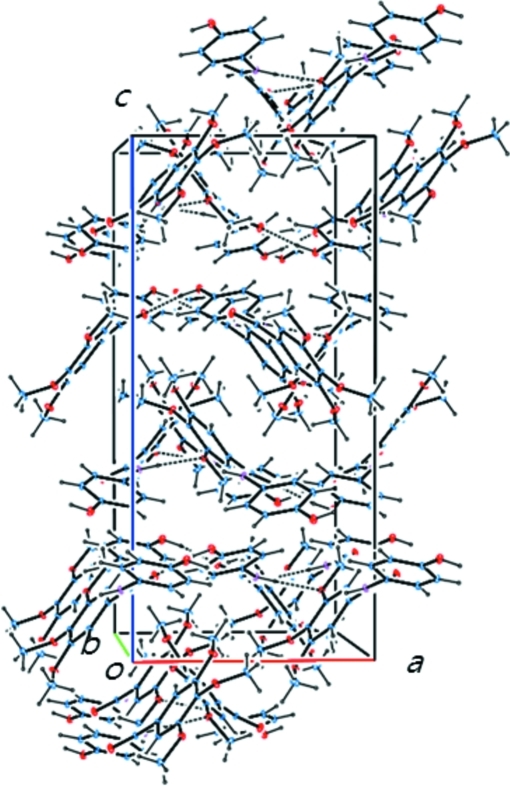
A packing diagram of the title compound, showing a two-dimensional network of molecules linked by intermolecular N—H···O and O—H···O hydrogen bonds (dashed lines).

## supplementary crystallographic information

### Comment

Tyrosinase, a multi functional copper containing enzyme, is widely distributed in the plant and animal kingdom. It is responsible for catalyzing *ortho*-hydroxylation of phenols and *ortho*-phenol oxidation to corresponding quinones (Kubo *et al.*, 2000). This enzyme was not only responsible for the browning of fruits and vegetables but also caused some dermatological problems such as flecks and melasma due to overproduction of melanin (Nerya *et al.*, 2004). Numerous potential tyrosinase inhibitors have been discovered from natural and synthetic sources, such as a kojic acid (Cabanes *et al.*, 1994), arbutin (Casanola-Martin *et al.*, 2006) and phenylthiourea (Thanigaimalai *et al.*, 2010). But some these inhibitors suffer from number of limitations, such as low activity and high toxicity. we have synthesized the title compound, (I), from the reaction of 3,4,5-trimethoxybenzoyl chloride and 4-aminophenol under ambient conditions. Herein, the crystal structure of (I) is described (Fig. 1).

The 3,4,5-trimethoxybenzoic acid moiety and 4-aminophenol group are essentially planar, with a mean deviation of 0.031 and 0.036 Å, respectively, from the corresponding least-squares plane defined by the ten and eight, respectively, constituent atoms. The dihedral angle between the benzene rings is 71.59 (4)°. The intermolecular N9—H9···O17^i^ and O16—H16···O8^ii^ [symmetry codes: (i) -*x* + 1/2, *y* - 1/2, *z*; (ii) -*x* - 1/2, *y* - 1/2, *z*; Table 1] hydrogen bonds allow to form an extensive two-dimensional network parallel to the *ab* plane (Fig. 2), which stabilizes the crystal structure.

### Experimental

The 3,4,5-trimethoxybenzoyl chloride and 4-aminophenol were purchased from Sigma Chemical Co. Solvents for organic synthesis were redistilled before use. All other chemicals and solvents were of analytical grade and were used without further purification. The title compound was prepared from the reaction of 3,4,5-trimethoxybenzoyl chloride (0.5 g, 1.0 mmol) and 4-aminophenol (0.4 g, 1.2 mmol) by simple substitution in THF(6 ml) with triethylamine (0.22 g, 1.2 mmol). The solvent was removed under reduced pressure. The mixture compound were purified by column chromatography on silica gel (2:1 dichloromethane/ethylacetate) to give the title compound (69%, m.p. 504 K). Colourless crystals of (I) were obtained from its ethanolic solution by slow evaporation of the solvent at room temperature.

### Refinement

Atoms H9 and H16 of the NH and OH groups were located in a difference Fourier map and refined freely [refined distances: N—H = 0.87 (2) Å and O—H = 0.88 (4) Å]. Other H atoms were positioned geometrically and refined using a riding model, with C—H = 0.93 or 0.96 Å, and with *U*_iso_(H) = 1.2*U*_eq_ (carrier C) for aromatic or 1.5*U*_eq_(carrier C) for methyl H atoms.

### Crystal data

**Table:**

C_16_H_17_NO_5_	*F*(000) = 1280
*M**_r_* = 303.31	*D*_x_ = 1.337 Mg m^−^^3^
Orthorhombic, *P**b**c**a*	Mo *K*α radiation, λ = 0.71073 Å
Hall symbol: -P 2ac 2ab	Cell parameters from 3298 reflections
*a* = 10.4280 (5) Å	θ = 2.7–25.0°
*b* = 13.4075 (6) Å	µ = 0.1 mm^−^^1^
*c* = 21.5565 (8) Å	*T* = 296 K
*V* = 3013.9 (2) Å^3^	Block, colourless
*Z* = 8	0.23 × 0.16 × 0.08 mm

### Data collection

**Table:**

Bruker SMART CCD area-detector diffractometer	*R*_int_ = 0.130
graphite	θ_max_ = 27.5°, θ_min_ = 2.7°
φ and ω scans	*h* = −13→10
14730 measured reflections	*k* = −14→17
3459 independent reflections	*l* = −11→27
2086 reflections with *I* > 2σ(*I*)	

### Refinement

**Table:**

Refinement on *F*^2^	Primary atom site location: structure-invariant direct methods
Least-squares matrix: full	Secondary atom site location: difference Fourier map
*R*[*F*^2^ > 2σ(*F*^2^)] = 0.054	Hydrogen site location: inferred from neighbouring sites
*wR*(*F*^2^) = 0.134	H atoms treated by a mixture of independent and constrained refinement
*S* = 0.97	*w* = 1/[σ^2^(*F*_o_^2^) + (0.0597*P*)^2^] where *P* = (*F*_o_^2^ + 2*F*_c_^2^)/3
3459 reflections	(Δ/σ)_max_ < 0.001
207 parameters	Δρ_max_ = 0.23 e Å^−^^3^
0 restraints	Δρ_min_ = −0.29 e Å^−^^3^

### Special details

**Table:**

Geometry. All s.u.'s (except the s.u. in the dihedral angle between two l.s. planes) are estimated using the full covariance matrix. The cell s.u.'s are taken into account individually in the estimation of s.u.'s in distances, angles and torsion angles; correlations between s.u.'s in cell parameters are only used when they are defined by crystal symmetry. An approximate (isotropic) treatment of cell s.u.'s is used for estimating s.u.'s involving l.s. planes.
Refinement. Refinement of *F*^2^ against ALL reflections. The weighted *R*-factor *wR* and goodness of fit *S* are based on *F*^2^, conventional *R*-factors *R* are based on *F*, with *F* set to zero for negative *F*^2^. The threshold expression of *F*^2^ > 2σ(*F*^2^) is used only for calculating *R*-factors(gt) *etc*. and is not relevant to the choice of reflections for refinement. *R*-factors based on *F*^2^ are statistically about twice as large as those based on *F*, and *R*- factors based on ALL data will be even larger.

### Fractional atomic coordinates and isotropic or equivalent isotropic displacement parameters (Å^2^)

**Table:**

	*x*	*y*	*z*	*U*_iso_*/*U*_eq_	
C1	0.09766 (17)	0.55474 (11)	0.38891 (8)	0.0352 (4)	
C2	0.09088 (17)	0.65316 (11)	0.36913 (8)	0.0352 (4)	
H2	0.0367	0.671	0.3367	0.042*	
C3	0.16570 (17)	0.72450 (11)	0.39825 (8)	0.0330 (4)	
C4	0.24487 (18)	0.69932 (11)	0.44779 (8)	0.0355 (4)	
C5	0.2478 (2)	0.60042 (11)	0.46863 (8)	0.0387 (4)	
C6	0.17580 (19)	0.52861 (12)	0.43820 (8)	0.0391 (5)	
H6	0.18	0.4624	0.451	0.047*	
C7	0.01230 (19)	0.48017 (12)	0.35789 (8)	0.0379 (4)	
O8	−0.09130 (14)	0.50472 (9)	0.33584 (7)	0.0557 (4)	
N9	0.05744 (18)	0.38648 (10)	0.35655 (8)	0.0435 (4)	
H9	0.139 (2)	0.3796 (14)	0.3652 (9)	0.058 (7)*	
C10	−0.00908 (19)	0.29931 (12)	0.33568 (8)	0.0376 (4)	
C11	0.06275 (19)	0.22529 (12)	0.30716 (8)	0.0407 (4)	
H11	0.149	0.236	0.2986	0.049*	
C12	0.0057 (2)	0.13529 (12)	0.29149 (8)	0.0421 (5)	
H12	0.0544	0.0854	0.273	0.051*	
C13	−0.1220 (2)	0.11949 (12)	0.30309 (8)	0.0417 (5)	
C14	−0.1944 (2)	0.19343 (13)	0.33081 (9)	0.0469 (5)	
H14	−0.2812	0.1831	0.3383	0.056*	
C15	−0.1375 (2)	0.28301 (13)	0.34741 (9)	0.0471 (5)	
H15	−0.1862	0.3324	0.3665	0.057*	
O16	−0.17129 (17)	0.02840 (9)	0.28685 (8)	0.0596 (5)	
H16	−0.251 (4)	0.023 (2)	0.2997 (14)	0.116 (12)*	
O17	0.16842 (13)	0.82370 (8)	0.38183 (6)	0.0436 (3)	
C18	0.0830 (3)	0.85680 (15)	0.33432 (11)	0.0723 (7)	
H18A	0.0951	0.9269	0.3274	0.108*	
H18B	−0.0039	0.8447	0.3469	0.108*	
H18C	0.1004	0.821	0.2967	0.108*	
O19	0.30929 (14)	0.77608 (8)	0.47554 (6)	0.0524 (4)	
C20	0.4380 (2)	0.76199 (16)	0.49453 (12)	0.0715 (7)	
H20A	0.4698	0.8224	0.5128	0.107*	
H20B	0.4896	0.745	0.4592	0.107*	
H20C	0.4419	0.709	0.5244	0.107*	
O21	0.32026 (16)	0.58220 (8)	0.52010 (6)	0.0580 (4)	
C22	0.3337 (3)	0.48202 (14)	0.54042 (11)	0.0732 (8)	
H22A	0.3862	0.4802	0.577	0.11*	
H22B	0.3733	0.4432	0.5083	0.11*	
H22C	0.2507	0.4549	0.5497	0.11*	

### Atomic displacement parameters (Å^2^)

**Table:**

	*U*^11^	*U*^22^	*U*^33^	*U*^12^	*U*^13^	*U*^23^
C1	0.0363 (11)	0.0263 (9)	0.0430 (10)	−0.0007 (7)	0.0014 (9)	−0.0003 (7)
C2	0.0353 (11)	0.0306 (9)	0.0396 (9)	0.0020 (7)	0.0002 (8)	0.0028 (7)
C3	0.0354 (10)	0.0234 (8)	0.0401 (9)	0.0021 (7)	0.0037 (8)	0.0033 (7)
C4	0.0389 (11)	0.0266 (8)	0.0409 (10)	−0.0013 (7)	−0.0026 (8)	−0.0033 (7)
C5	0.0446 (12)	0.0319 (9)	0.0397 (10)	0.0024 (8)	−0.0066 (9)	0.0024 (7)
C6	0.0453 (12)	0.0249 (9)	0.0470 (11)	0.0020 (7)	−0.0027 (9)	0.0030 (7)
C7	0.0368 (12)	0.0307 (10)	0.0462 (10)	−0.0018 (7)	−0.0028 (9)	0.0046 (7)
O8	0.0433 (9)	0.0392 (7)	0.0847 (11)	−0.0009 (6)	−0.0179 (8)	0.0071 (6)
N9	0.0376 (10)	0.0280 (8)	0.0648 (11)	−0.0005 (7)	−0.0098 (9)	−0.0062 (6)
C10	0.0405 (12)	0.0289 (9)	0.0434 (10)	−0.0031 (7)	−0.0049 (9)	−0.0007 (7)
C11	0.0345 (11)	0.0392 (10)	0.0484 (11)	−0.0028 (8)	−0.0007 (9)	−0.0019 (8)
C12	0.0455 (12)	0.0350 (10)	0.0458 (11)	−0.0004 (8)	0.0019 (9)	−0.0071 (8)
C13	0.0486 (13)	0.0320 (10)	0.0445 (10)	−0.0083 (8)	0.0010 (10)	−0.0025 (7)
C14	0.0375 (12)	0.0407 (11)	0.0626 (13)	−0.0059 (8)	0.0056 (10)	−0.0013 (9)
C15	0.0431 (12)	0.0328 (10)	0.0653 (13)	−0.0001 (8)	0.0035 (11)	−0.0067 (8)
O16	0.0579 (11)	0.0425 (8)	0.0784 (11)	−0.0177 (7)	0.0125 (9)	−0.0186 (7)
O17	0.0488 (9)	0.0251 (6)	0.0571 (8)	−0.0018 (5)	−0.0083 (7)	0.0092 (5)
C18	0.0845 (19)	0.0405 (12)	0.0918 (17)	−0.0007 (11)	−0.0379 (15)	0.0227 (11)
O19	0.0577 (10)	0.0328 (7)	0.0666 (9)	−0.0043 (6)	−0.0216 (7)	−0.0053 (6)
C20	0.0641 (17)	0.0598 (14)	0.0906 (17)	−0.0157 (11)	−0.0370 (15)	0.0139 (12)
O21	0.0823 (12)	0.0352 (7)	0.0564 (8)	−0.0025 (7)	−0.0279 (8)	0.0102 (6)
C22	0.105 (2)	0.0445 (13)	0.0700 (15)	0.0016 (12)	−0.0323 (16)	0.0168 (10)

### Geometric parameters (Å, °)

**Table:**

C1—C6	1.384 (2)	C12—C13	1.371 (3)
C1—C2	1.389 (2)	C12—H12	0.93
C1—C7	1.496 (2)	C13—O16	1.371 (2)
C2—C3	1.385 (2)	C13—C14	1.382 (3)
C2—H2	0.93	C14—C15	1.386 (2)
C3—O17	1.3766 (17)	C14—H14	0.93
C3—C4	1.391 (2)	C15—H15	0.93
C4—O19	1.3670 (19)	O16—H16	0.88 (4)
C4—C5	1.400 (2)	O17—C18	1.428 (2)
C5—O21	1.364 (2)	C18—H18A	0.96
C5—C6	1.386 (2)	C18—H18B	0.96
C6—H6	0.93	C18—H18C	0.96
C7—O8	1.225 (2)	O19—C20	1.416 (3)
C7—N9	1.342 (2)	C20—H20A	0.96
N9—C10	1.432 (2)	C20—H20B	0.96
N9—H9	0.87 (2)	C20—H20C	0.96
C10—C15	1.381 (3)	O21—C22	1.420 (2)
C10—C11	1.387 (2)	C22—H22A	0.96
C11—C12	1.387 (2)	C22—H22B	0.96
C11—H11	0.93	C22—H22C	0.96
			
C6—C1—C2	120.41 (15)	C11—C12—H12	119.8
C6—C1—C7	121.60 (15)	O16—C13—C12	117.10 (17)
C2—C1—C7	117.88 (16)	O16—C13—C14	123.00 (19)
C3—C2—C1	119.24 (16)	C12—C13—C14	119.90 (16)
C3—C2—H2	120.4	C13—C14—C15	119.97 (19)
C1—C2—H2	120.4	C13—C14—H14	120
O17—C3—C2	124.22 (15)	C15—C14—H14	120
O17—C3—C4	114.81 (14)	C10—C15—C14	120.31 (17)
C2—C3—C4	120.97 (14)	C10—C15—H15	119.8
O19—C4—C3	116.41 (14)	C14—C15—H15	119.8
O19—C4—C5	124.17 (16)	C13—O16—H16	110.2 (18)
C3—C4—C5	119.28 (15)	C3—O17—C18	118.15 (14)
O21—C5—C6	124.09 (14)	O17—C18—H18A	109.5
O21—C5—C4	116.28 (15)	O17—C18—H18B	109.5
C6—C5—C4	119.60 (16)	H18A—C18—H18B	109.5
C1—C6—C5	120.43 (15)	O17—C18—H18C	109.5
C1—C6—H6	119.8	H18A—C18—H18C	109.5
C5—C6—H6	119.8	H18B—C18—H18C	109.5
O8—C7—N9	123.53 (17)	C4—O19—C20	119.47 (15)
O8—C7—C1	121.22 (15)	O19—C20—H20A	109.5
N9—C7—C1	115.25 (17)	O19—C20—H20B	109.5
C7—N9—C10	126.95 (18)	H20A—C20—H20B	109.5
C7—N9—H9	115.8 (13)	O19—C20—H20C	109.5
C10—N9—H9	116.8 (13)	H20A—C20—H20C	109.5
C15—C10—C11	119.46 (16)	H20B—C20—H20C	109.5
C15—C10—N9	122.81 (16)	C5—O21—C22	118.36 (14)
C11—C10—N9	117.50 (17)	O21—C22—H22A	109.5
C12—C11—C10	119.92 (18)	O21—C22—H22B	109.5
C12—C11—H11	120	H22A—C22—H22B	109.5
C10—C11—H11	120	O21—C22—H22C	109.5
C13—C12—C11	120.43 (17)	H22A—C22—H22C	109.5
C13—C12—H12	119.8	H22B—C22—H22C	109.5

### Hydrogen-bond geometry (Å, °)

**Table:**

*D*—H···*A*	*D*—H	H···*A*	*D*···*A*	*D*—H···*A*
N9—H9···O17^i^	0.87 (2)	2.18 (2)	3.029 (2)	165.8 (17)
O16—H16···O8^ii^	0.88 (4)	1.84 (4)	2.710 (2)	172 (3)

Symmetry codes: (i) −*x*+1/2, *y*−1/2, *z*; (ii) −*x*−1/2, *y*−1/2, *z*.
